# How the Interval between Prime and Boost Injection Affects the Immune Response in a Computational Model of the Immune System

**DOI:** 10.1155/2012/842329

**Published:** 2012-09-11

**Authors:** F. Castiglione, F. Mantile, P. De Berardinis, A. Prisco

**Affiliations:** ^1^Institute for Computing Applications “M. Picone”, National Research Council of Italy, via dei Taurini 19, 00185 Roma, Italy; ^2^Institute of Genetics and Biophysics “A. Buzzati Traverso”, National Research Council of Italy, via Pietro Castellino 111, 08013 Naples, Italy; ^3^Institute of Protein Biochemistry National Research Council of Italy, via Pietro Castellino 111, 08013 Naples, Italy

## Abstract

The immune system is able to respond more vigorously to the second contact with a given antigen than to the first contact. Vaccination protocols generally include at least two doses, in order to obtain high antibody titers. We want to analyze the relation between the time elapsed from the first dose (priming) and the second dose (boost) on the antibody titers. In this paper, we couple *in vivo* experiments with computer simulations to assess the effect of delaying the second injection. We observe that an interval of several weeks between the prime and the boost is necessary to obtain optimal antibody responses.

## 1. Introduction

Immunological memory, defined as the capacity of the immune system to respond more vigorously to the second contact with a given antigen than to the first contact, is the basis of the persistent protection afforded by the resolution of some infections and is the goal of vaccination. Memory is a system-level property of the immune system, which arises from the increase in the frequency of antigen specific B and T cells as well as from the differentiation of antigen specific lymphocytes into memory cells, which are able to respond faster to antigen and to self-renew [1–3].

The protection afforded by vaccines currently in use correlates well with the magnitude of the antibody response. The persistence of antigen-specific antibody titers over a protective threshold and the ability to exhibit a “recall response” to eencounter with antigen have long been the only measurable correlates of vaccine “take” and immune memory. However, these methods for the evaluation of immune memory suffer from the disadvantage of relying on long-term monitoring of the immune response. Thus, optimizing the vaccination schedule to obtain high and persisting antibody titers, an important step in the development of novel vaccines and immunotherapies, is a long trial and error process [4, 5].

The magnitude of the immune response can usually be increased by multiple administrations of vaccine; the notable exception being represented by virus-vectored vaccines and whereby immunity to the viral capsid induced by the first dose prevents cell infection by subsequent doses.

When a new prototype vaccine is tested for the first time *in vivo*, the injection schedule is designed empirically, using a combination of immunological knowledge, previous experience, and practical constraints, and it is refined on the basis of the observed immunological responses and protection. However, *in vivo* experimentation poses practical limits to the number of different immunization schedules that can be tried to find the protocol that maximizes the antibody titer, while minimizing the number of doses. Thus, *in silico* simulations of the kinetics of the antibody response can be useful to generate predictions, that can then be tested experimentally, and to generate novel hypotheses on early correlates of immune memory.

The vaccine used to generate the experimental data reported in this study and described in Section 2, namely-(1-11)E2, consists of “virus-like particles” formed by a domain of the bacterial protein E2 that is able to self-assemble into a 60-mer peptide [6]. Each particle displays on its surface 60 copies of peptide “DAEFRHDSGYE,” corresponding to the first 11 N-terminal residues of beta-amyloid, a peptide that forms aggregates in the brain of Alzheimer's disease patients.

A single “prime” dose of the (1-11)E2 vaccine induces measurable titers of anti-beta-amyloid antibodies in all treated mice, and in 4/5 mice that received a “boost” dose 6 months later, we observed a clear memory response, namely, a fast rise of anti-beta-amyloid antibody titers to a peak serum concentration between 1 and 7 mg/mL.

Studies performed in transgenic mouse models of Alzheimer's disease have demonstrated that antibodies against beta-amyloid are able to reduce plaques and improve cognition (reviewed in [7–10]. In mouse models as well as in clinical trials in Alzheimer's disease patients, induction of a high titer of anti-beta-amyloid antibodies correlates with the therapeutic efficacy of vaccination [10, 11].

In this study, the effect of the time delay between the first and the second injection of antigen on the peak antibody titer is explored in an computer model of the immune system response. 

## 2. Materials and Methods 

### 2.1. Animals

 BALB/c mice were obtained from Charles River Laboratory, Italy. Ethics Committee of the institution within which the work was undertaken have approved the protocols involving mice and these conform to the provisions of the Declaration of Helsinki and Italian National Guidelines for animal use in research.

### 2.2. Generation of Virus-Like Particles (VLP) (1-11)E2

 Synthetic complementary oligonucleotides encoding the sequence 1–11 (sequence DAEFRHDSGYE) of beta-amyloid were cloned into the pETE2DISP vector cut with NcoI and XmaI, to obtain plasmid pET(1-11)E2. Successful construction of the plasmid was confirmed by DNA sequence analysis. (1-11)E2 VLP was produced and characterized as previously described [5].

### 2.3. Immunizations

 Mice were immunized intraperitoneally with 200 *μ*L of a 1 : 1 mixture of antigen and adjuvant. Complete Freund's Adjuvant (CFA) was used in the first injection, and Incomplete Freund's Adjuvant (IFA) in the second one. Each mouse received an amount of antigen carrying 6 *μ*g of the beta-amyloid epitope. Blood was collected at indicated time points, and ELISA was performed on serum.

### 2.4. Enzyme-Linked Immunosorbent Assay (ELISA)

 Wells of a 96-well Nunc Immunoplate were coated with streptavidin at 37°C over night until complete evaporation. Wells were blocked with 0.5% bovine serum albumin in 20 mM TrisHCl pH 7.3, and 120 mM NaCl, incubated with 50 ng biotinylated peptide, incubated with mouse sera diluted in 0.25% bovine serum albumin, 20 mM TrisHCl pH 7.3, 0.5 M NaCl, 0.05% Tween 20, and detected with anti-mouse IgG peroxidase conjugate (SIGMA A-2554).

All incubations were carried out for 1 hr at 37°C, and after each step wells were washed twice with Elisa wash buffer (EWB) (20 mM TrisHCl pH 7.3, 130 mM NaCl, 0.05% Tween 20) and once with Tris buffered saline (TBS) (20 mM TrisHCl pH 7.3, 0.5 M NaCl). Wells were incubated for 45 min at room temperature with 0.4 mg mL^−1^ O-phenylenediamine dihydrochloride dissolved in 30 mM citric acid, 70 mM Na_2_HPO_4_, 0.8 mM H_2_O_2_. Absorbance was read at 492 nm, after blocking color development was blocked with 0.8 M sulfuric acid.

Each serum was tested against synthetic peptides 1–11 of beta-amyloid (the synthetic peptide 23–29 of beta-amyloid was used as a negative control). Titer of a serum was defined as the highest dilution yielding an absorbance value equal to twofold of the background value obtained against an irrelevant antigen.

### 2.5. The Computational Model

 The *in silico* experiments are performed by a computational model of the immune system [12] that uses binary strings to represent the *binding site* of cells and molecules (i.e., lymphocytes receptors, BCRs, TCRs, Major Histocompatibility Complexes MHC, antigen peptides and epitopes, immunocomplexes IC, etc.).

The model is based on the agent-based modeling (ABM) paradigm, in that all entities are individually represented [13, 14] as in cellular automata models [15]. It includes the major classes of cells of the lymphoid lineage, that is, T helper lymphocytes, cytotoxic T lymphocytes, B lymphocytes, antibody-producer plasma cells, and natural killer cells (NK) and some of the myeloid lineage, that is, macrophages (M*ϕ*) and dendritic cells (DC). These entities cooperate following a set of algorithms (or logical rules) carrying out the different phases of the immune recognition and response to a generic pathogen. In particular, the model takes into account phagocytosis, antigen presentation, cytokine release, cell activation from inactive or anergic states to active states, cytotoxicity, and antibody secretion. The model simulates a simplified form of innate immunity and a more elaborate form of adaptive immunity, including both humoral and cytotoxic immune responses [16].

In the model, a single human lymph node (or a portion of it) is mapped onto a three-dimensional ellipsoid Cartesian lattice. The primary lymphoid organs thymus and bone marrow are modeled apart: the thymus [17] is implicitly represented by the positive and negative selection of immature thymocytes before they enter into the lymphatic system, while the bone marrow generates already mature B lymphocytes. Hence, only immunocompetent lymphocytes are represented on the primary lymphoid organ modeled.

This computational model can be seen as a collection of working assumptions or theories, most of which are regarded as established immunological mechanisms. In details, the model includes: the clonal selection theory of Burnet [18]; the idiotypic network theory of Jerne [19]; the clonal deletion theory (i.e., thymus education of T lymphocytes, [20]); the hypermutation of antibodies [21]; the danger theory of Matzinger [22]; the replicative senescence of T cells, or the Hayflick limit (i.e., a limit in the number of cell divisions, [23]); T-cell anergy [24]; Ag-dose-induced tolerance in B cells [25]. These features can be selectively toggled on or off, allowing for general investigations of immunological hypothesis. Moreover, other specific biological processes can be added to the model with relatively little effort. For example, customizations of the basic model have been used to simulate different phenomena ranging from viral infection (e.g., HIV, EBV [26, 27]) to type I hypersensitivity [28] and cancer [29, 30].

A simulated time step is roughly equivalent to eight hours. The interactions among the cells determine their functional behavior (Table 1). Interactions are coded as probabilistic rules defining the transition of each cell entity from one state to another. Each interaction requires the involved cellular entities to be in a specific state out of a set of possible states (e.g., naïve, active, resting, duplicating) that is dependent on the cell type. Once these conditions are fulfilled, the interaction is driven by a probability that is directly related to the effective level of binding between ligands and receptors. 

**Figure 1:**
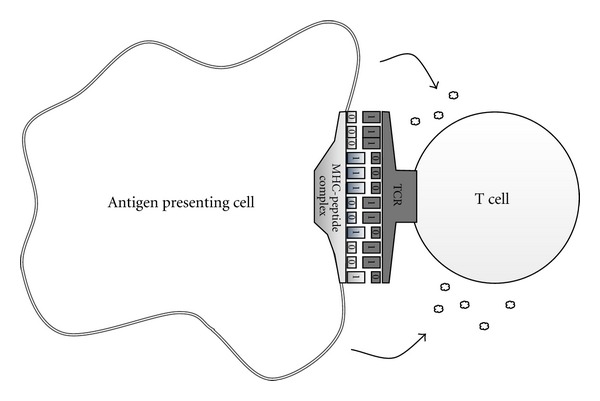
Molecular affinity is calculated on the basis of the Hamming distance of the binary strings representing the binding sites of the interacting entities. In the figure a T lymphocyte receptor binds the MHC-peptide complex of an antigen presenting cell.

 Strings of 0s and 1s are used to represent specificity elements like receptors and other molecular binding specificities (see Figure 1). The length of this string is specified as a parameter *ℓ*. Two bit-strings complement each other (or are a perfect match) if every 0 in one corresponds to a 1 in the other and conversely. More generally, an *m*-bit match is defined as a pair where exactly *m* bits complement each other. Therefore, in order to compute the binding probability, we first define the function *h*(*a*, *b*) giving us the number of matching bits between two strings *a* and *b* (i.e., the Hamming distance in the space of the bit-strings). Then, we define the function *α*(*m*) as the affinity of an *m*-bit match. To ensure that perfect matches prevail over imperfect ones, we set *α*(*ℓ*) to a high value and *α*(*m*) (with *m* < *ℓ*) to lower values. To specify the vector *α*, one method is to specify it directly by simply listing out its components. Another method uses the additional parameter arguments $\underline{m}$, that is, the minimum match allowed, $\underline{a}=\alpha (\underline{m})$, that is, the minimum level of affinity, and *δ*_*α*_ a parameter specifying the gain in affinity proportional to a one bit more match, to calculate in the following way: (i) using the parameter $\underline{m}$, set $\alpha \left(\underline{m}\right)=\underline{a}$ whereas for $m<\underline{m}$ set *α*(*m*) to 0 (this provides a level below which binding cannot occur); (ii) the increase of strength on increasing a match by one bit is set to be the inverse of the ratio of number of clones with match *m* + 1 and *m* multiplied by the parameter *δ*_*α*_. In formula,

(1) $$\begin{array}{c} \frac{\alpha \left(m+1\right)}{\alpha \left(m\right)}=\frac{\delta_{\alpha }\left(\begin{array}{c} \ell \\ m \end{array}\right)}{\left(\begin{array}{c} \ell \\ m+1 \end{array}\right)}. \end{array}$$

This allows to set the lower end value of *α*(*m*) and the steepness of its increase as the number of matching bits is incremented. It is usually more convenient than supplying the *α* vector directly. Generally, it is advisable to set $\underline{m}$ somewhat close to *ℓ* bits in order to restrict the range of allowable matches to a few bits, so that the number of antibodies raised in response to a given antigen remains manageable.

Unlike the many immunological models, the present one not only simulates the cellular level of the intercellular interactions but also the intra-cellular processes of antigen uptake and presentation. Both the cytosolic and endocytic pathways are modeled. In the model, endogenous antigen is fragmented and combined with MHC class I molecules for presentation on the cell surface to CTLs receptors, whereas the exogenous antigen is degraded into smaller parts (i.e., peptides), which are then bound to MHC class II molecules for presentation to the THs receptors (Table 1). The affinity among MHC molecules and the antigen peptides is computed in a slightly different manner than those between cell receptors and antigenic epitopes. Firstly, the match is computed over half bit string; secondly, there is no minimum match. The affinity value between two half strings whose match is *m*, for  all  *m* = 0,…, *ℓ*/2, is defined as

(2) $$\begin{array}{c} \beta \left(m\right)={\left(\frac{1}{2}\right)}^{\ell /2-m}. \end{array}$$

The function *β*(*m*) represents the probability that a peptide with match *m* to the MHC molecule binds and is presented alongside with it on the cell surface for subsequent TCR recognition.

While macroscopic entities like cells are individually represented (i.e., they are considered as agents), low-molecular, weight molecules, such as interleukins or chemokines, are represented in terms of their concentration. The corresponding dynamics is modeled by the following parabolic partial differential equation that describes a uniform diffusion process with the addition of a degradation term that takes into account the finite half-life of molecules:

(3) $$\begin{array}{c} \frac{\partial c}{\partial t}=D\nabla^{2}c-\lambda c+s\left(x,t\right), \end{array}$$

where *c* = *c*(*x*, *t*) is the concentration of chemokines, *s*(*x*, *t*) is the source term, *D* is the diffusion coefficient, and *λ* = ln⁡⁡2/*τ* where *τ* is the half-life. We assume *D* = 3000 *μ*m^2^/min and *τ* = 3 hrs for all chemokines [31, 32]. Differences in cell mobility also are taken into account. TH cells are the fastest with an average velocity of 11 *μ*m/min, followed by B cells with 6 *μ*m/min and DC with a velocity of 3 *μ*m/min [32].

The rules listed in Table 1 are executed for each time step. The stochastic execution of these rules, as in a Monte Carlo methods, produces a logical causal/effect sequence of events culminating in the immune response and development of immunological memory. The starting point of this series of events is the injection of antigen (the priming).

The system is designed to maintain a steady state of the global population of cells (*homeostasis*) if no infection is applied. This is achieved by modeling the birth/death process as a mean reverting process of the type:

(4) $$\begin{array}{c} \frac{dx_{i}\left(t\right)}{dt}=\frac{\log_{2}}{\tau_{i}}\left(x_{i}\left(0\right)-x_{i}\left(t\right)\right)+\sigma \left(t\right), \end{array}$$

where *x*_*i*_(*t*) is the population *i* at time *i*, *τ*_*i*_ is the specific half-life parameter, and *σ*(*t*) is a Gaussian random noise.

Initially the system is naïve in the sense that there are neither *T* and *B* memory cells nor plasma cells and antibodies. The various steps of the simulated immune response depends on what is actually injected, for example, a recombinant virus or bacteria.

The model contains a number of parameters whose value has been determined as follows. These parameters can be classified into three categories: (i) unknown values or free parameters, which are set after a tuning procedure that begins with an initial estimation of their values and iteratively improves the results of the simulations by small modifications of the parameters; (ii) parameters that correspond to the initial conditions of the system and that determine the problem under investigation; (iii) parameters whose value is well known and available from immunology literature.

Given the initial condition represented by the simulated volume determining the number of cells populating the space according to known leukocyte formulas, the model runs in a metastable state assured by homeostasis. In absence of antigenic stimulus, the populations of immune cells randomly fluctuates around the average values given. Upon an antigenic challenge performed by injecting a certain amount of a pathogen, the system moves away from the metastable state to recognize the insulting molecules and to mount an immune response that may or not include the deployment of both the humoral and the cytotoxic artillery. Once the antigen is cleared, the system goes back to an equilibrium state that is not the same as before as it contains a shift in the system specificity amounting to the immune memory. This memory allows for a faster and stronger reaction to a later encounter of the same (or similar) pathogen.

**Figure 2:**
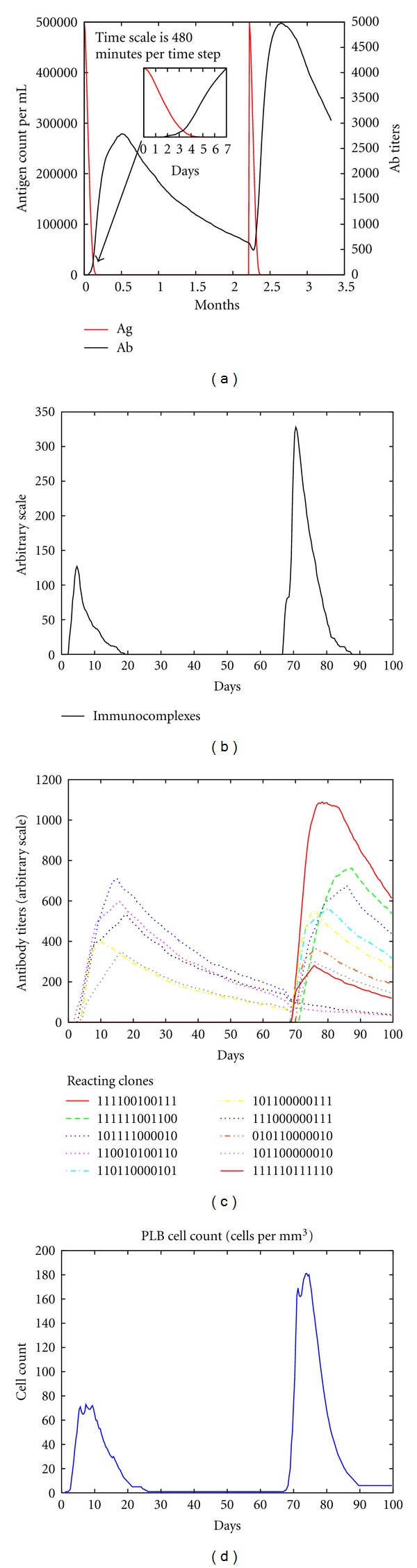
The virtual experiments are conducted priming at day zero and later after a certain time interval. In this case the boost has been performed after about ten weeks. While the antibodies are produced by plasma cells derived by expanding clones of B cells, the injected antigen is cleared and immunocomplexes are formed. The secondary immune response to the boost is stronger and faster than the response to the priming because of the immunological memory (not shown).

Figure 2 shows this dynamics as an example of a typical immunization experiment consisting in injecting at day zero and about ten weeks after a generic immunogenic substance as a vaccine. The result of the priming is that the antigen is cleared in about four days (panel up-left) as the antibodies elicited peak within the second and the third week (bottom-left panel). The different specificities (i.e., binary strings) of the antibodies elicited are shown in the same figure. The figure also shows the corresponding antibody-producing plasma cells (bottom-right panel) and the immunocomplexes titer (up-right panel) consisting of antigen clotted with antibodies.

 Whereas the immunogenicity of the injected substance is the main responsible for the immune response, a secondary but not less important factor is the timing. Indeed, as anticipated above, the question investigated here is what is the optimal timing for boosting in terms of higher antibody titers. Intuitively, one expects a window of optimality since a too close boost does not elicit a strong memory as it simply add, (and compete for resources) to the prime, whereas an overly delayed boost may fail to wake up the memory simply because it already faded away. Computer simulations allow to easily broadening the search for the optimality, something that would be costly and time consuming with animal models.

## 3. Validating the Model against the Experimental Dataset

Before use, the simulator needs to be validated against the specific experimental data available and described in Section 2. Interestingly, matching experimental data was not straightforward. Indeed, the first set of simulations did not yield reasonable fit with the data indicating that the model was lacking of some specific mechanism.

In particular, the model failed to reproduce a correct kinetic for both antigen clearance and antibody expansion (it goes without saying that the two issues are connected) as we obtained faster than experimentally observed rates. Discussions pointed us to identify a mechanism of vaccine delivery that was missing in the computational model and could account for the divergence observed. Therefore, in order to correct this inconsistency, we implemented two mechanism: (i) one to implement what is called the “depot effect,” that is, the gradual release of the vaccine so as to cover a long period of antigen exposure, and (ii) a mechanism accounting for immunocomplexes dissociation actually providing a further longer exposition time to the injected vaccine.

**Figure 3:**
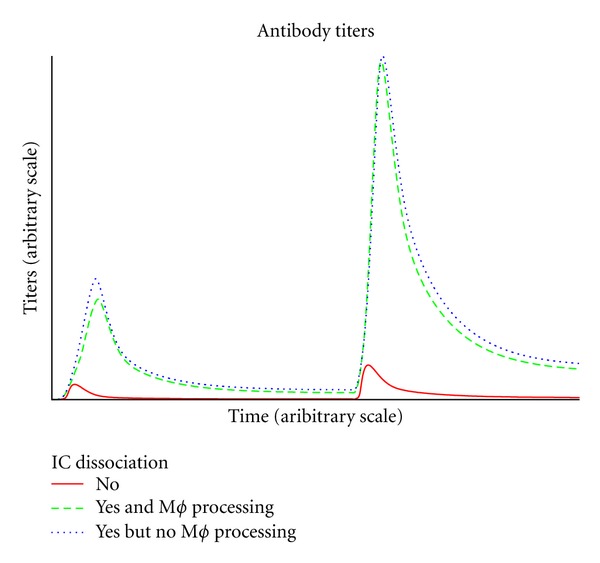
Dissociation of immunocomplexes and successive phagocytosis and presentation by APCs of the antigenic molecules increases vaccine persistence hence increasing the magnitude of the immune response.

The modified model incorporating these two effects effectively increased the targeted adherence to the experimental data. Since the depot effect resulted in a minor difference, we show hereafter the effects of implementing the dissociation of immunocomplexes on the simulation outcome. Note that the overall expected effect of the antigen-antibody compound dissociation is to have a longer exposition to the antigen and also a better affinity maturation since weak binders have a higher dissociation rate. Specifically, the instability of immunocomplexes (ICs) favors re-ingestion of the immunogenic peptides by antigen presenting cells (APCs) and representation to specific lymphocytes, who, on their side, opt for higher affinity ones. See Figure 3 to compare the antibodies responses in three different cases: without IC dissociation, with IC dissociation but no direct ingestion and following presentation of IC by macrophages and with both IC dissociation but no competing mechanism of IC elimination by macrophages. We can see that without IC dissociation, the antibody titers are low compared to the case of higher antigen-antibody instability whereas the effect of a direct ingestion and following presentation of IC by macrophages does not account for the same big effect but nevertheless shows that IC ingestion by M*ϕ* actually represents a suboptimal situation compared to the “neat” IC dissociation because of the waste of antibodies bound to the antigen in the complexes that are effectively thrown away by macrophages upon ingestion. 

**Figure 4:**
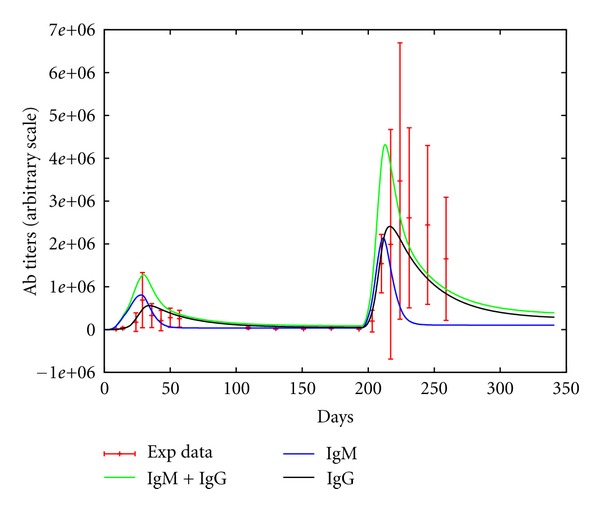
Comparison with mice data: antibody (IgG) titers as average of four mice experiments with relative standard deviation. Mice received a prime injection at day 0 and a boost six months later.

 After these modifications the simulator showed titers that are comparable to that observed in real data.  Figure 4 show the fit with mice data calculated as average of four mice experiments. Error bars show the standard deviation of IgG antibodies receiving a vaccine priming at day zero and a boost six months later. The solid line in Figure 3 show a good agreement of the simulated mice with the experimental data.

This data set allowed to fine tune the parameters of the simulator. Further experiments have been performed afterwards to investigate the relationships among the prime-boost time distance and the magnitude of the immune response measured as IgG antibody titers. This is show in the next section.

## 4. Results

 In order to investigate the relationship between the interdose delay and the immune response, we have performed a set of virtual experiments by running the simulation with different initial conditions. In particular, we injected the antigen at time step *t*_1_ = 0 and successively at *t*_2_. We performed simulations for *T* time steps, corresponding to about *T*/3 days of real life. The delay *δ*_*t*_ = *t*_2_ − *t*_1_ is the free variable of the experiment, whereas the outcome is the differences in the amount of antibodies produced to the prime and the boost vaccination. More specifically, we call *ab*(*t*) the antibody titers at time *t*, *m*_1_ = max⁡⁡{*ab*(*t*) : *t*_1_ ∈ [*t*_1_, *t*_2_)} the maximum level of *ab* relative to the injection of antigens at time *t*_0_ (i.e., the prime injection), and analogously *m*_2_ = max⁡⁡{*ab*(*t*) : *t* ∈ [*t*_2_, *T*]} the maximum level of IgG antibodies relative to the injection of antigens at time *t*_2_ (i.e., the boost injection). We can assume that *m*_1_ ≤ *m*_2_ since the injected antigen is the same for the two injections and, therefore, the immune memory is such that the second immune response is faster and stronger than the first [33, 34].

We call Δ_*ab*_ = *m*_2_ − *m*_1_ the differences in the peak values of antibody titers during the two responses. Since *t*_1_ is fixed, *t*_1_ = 0 and *m*_1_ and *m*_2_ both depend on the time of the second injection *t*_2_, we have that *δ*_*t*_ = *t*_2_, *m*_2_ ≡ *m*_2_(*t*_2_) and Δ_*ab*_ ≡ Δ_*ab*_(*t*_2_).

**Figure 5:**
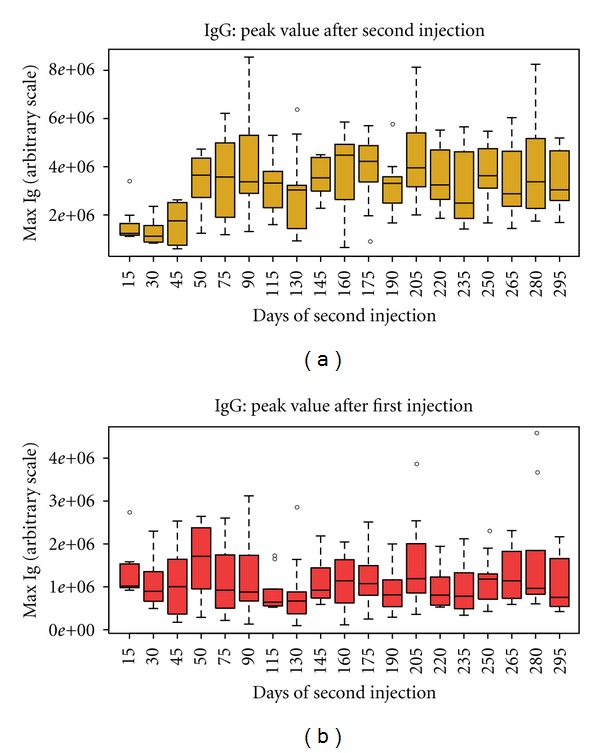
The lower panel shows that *m*_1_ is trivially independent of *t*_2_ whereas the upper panel showing *m*_2_ tells that, overall, there exists an optimal timing for the boost that is greater than 45 days.

In Figure 5, we show a boxplot to compare *m*_1_(0) and *m*_2_(*t*_2_) for different values of *δ*_*t*_ = *t*_2_. This has been computed averaging over 20 simulations of 10 micro liters of volume. The lower panel of that figure shows that, apart from large stochastic fluctuation, *m*_1_(*t*_2_) = const, that is, it is independent of *t*_2_, whereas the upper panel showing *m*_2_(*t*_2_) tells that, overall, there exists an optimal timing for the boost that is greater than 45 days. 

**Figure 6:**
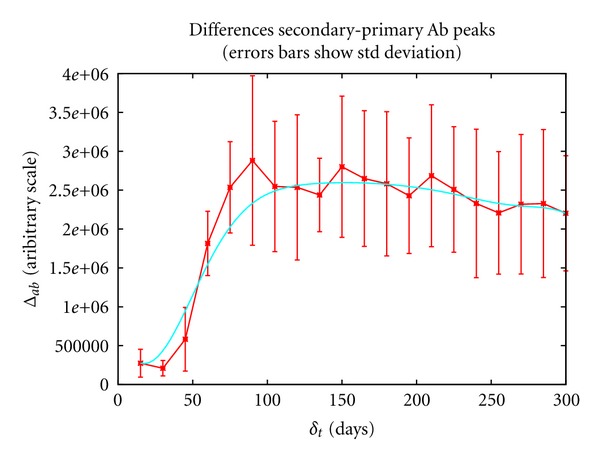
When the boost is given in the first month after the prime, our measure of the efficiency of the boost, Δ_*ab*_(*δ*_*t*_), is quite low whereas it increases when the second dose is given 45 to 90 days after the prime.

The same information is better displayed in Figure 6 that plots Δ_*ab*_(*δ*_*t*_) as a function of *δ*_*t*_ = *t*_2_. In particular, an interval of several weeks between the prime and the boost is necessary to obtain an optimal humoral response, as hypothesized and reported in experimental studies. Indeed, when the boost is given in the first month after the prime, the difference between the peaks of the secondary and primary responses, that is a measure of the efficiency of the boost, is quite low. The boost efficiency increases when the second dose is given 45 to 90 days after the prime, whereas further delaying the boost does not improve the secondary antibody peak.

## 5. Discussion

 Optimizing prime-boost regimens is key to developing novel vaccines. What is the optimal time for boosting is a fundamental question that remains unanswered [4]. It has been suggested that an interval of at least 2-3 months between the prime and the boost is necessary to obtain optimal responses, as memory T cells with high proliferative potential do not form until several weeks after the first immunization, and memory *B* cells have to go through the germinal center reaction and take several months to develop [4].

Immunization schedules are designed empirically and are then refined on the basis of the observed immunological responses and protection. In some instances, different countries that implement the same vaccine in their national immunization programs use different schedules [35, 36].

The United States Advisory Committee on Immunization Practices (ACIP) publishes each year a recommended immunization schedules for licensed vaccines, to reflect current recommendations [37, 38]. For individuals whose vaccinations have been delayed, catch up schedules and minimum intervals between doses are indicated [37]. For most vaccines currently in use, the minimum recommended interval between dose 1 and dose 2, for children, is 4 weeks, however, for some vaccines a minimum interval of 8 weeks, 3 months, or 6 months is recommended [37].

In preclinical experimentation of prototype vaccines, on the other hand, shorter intervals between doses are often used, to obtain a rapid rise in antibody titers above protective values. In the case of vaccination against beta-amyloid in mouse models of Alzheimer's disease, a schedule that has been used with a variety of prototype vaccines involves doses at day 0, 2 weeks and 4 weeks, and monthly doses thereafter. When multiple doses are administered within a short timeframe, understanding the contribution of each dose to the peak antibody titer can be practically impossible.

In this study we have analyzed the effect of the interval between prime and boost injection on the antibody response in a computational model of the immune system.

We have shown that in the computational model an interval of several weeks between the prime and the boost is necessary to obtain optimal responses, as hypothesized and reported for real immune responses. In particular, in the simulations, when the boost is given in the first month after the prime, the difference between the peaks of the secondary and primary responses, our measure of the efficiency of the boost, is low. The boost efficiency increases when the second dose is given 45 to 90 days after the prime, whereas further delaying the boost does not improve the secondary antibody peak (simulations of boosts administered up to 300 days after the prime are shown in Figure 6).

Thus, the computational model displays the qualitative features of real immune responses, and it can be useful to understand which component of the immune system is in charge for the time-dependent differences in boost efficacy that are observed *in vivo*. Interestingly, the efficacy of the boost does not parallel the number of T helper cells and B cells. In the model, the number of T and B cells increases after the prime, as cells are activated and duplicate. Cell numbers then decline, as a consequence of cell death. Thus, at day 15 there are more T or B cells than at day 90. Interestingly, also memory T cells are more abundant at the 15 and 45 time point than at later time points, revealing that the better memory response obtained at later time points is not correlated to higher numbers of memory T cell. On the contrary, the boost is optimal at a time point when the populations generated by the prime, in particular, activated cells, duplicating cells, and also memory cells, have all contracted. The T and B cells that are present in the system at late time points after the prime are qualitatively different from earlier cells. It is important here to emphasize that, in the model, a memory cell is a cell that, having been activated by antigen, has increased its average lifespan. Further encounters with antigen lead to further increases in the lifespan. Thus, memory cells are not all equal in their proliferative potential, and the memory of the system matures over time, as cells with high proliferative capacity are generated. This model, therefore, demonstrates that cell populations dynamics, and a simple assumption, namely the fact that a “survival signal” is received by memory cells at each encounter with antigen, are sufficient to reproduce the need for an optimal delay between prime and boost, observed *in vivo.*

On the other hand, different vaccines are known to have different requirements with respect to the minimum interval between doses. The simulations reported in this study refer to a “generic vaccine,” and the time scales that were obtained, which are quite realistic, anyway do not refer to a specific vaccine, although parameters have been set to fit data obtained with a nonreplicating protein antigen, namely, virus-like particle (1-11)E2 (6). The computational model can be useful to explore the role of different features of the primary response on the optimal time point for boost, and on boost efficiency, at a set time point.

A deeper analysis of the overall system dynamics is currently underway to pinpoint which immune component is in charge for the observed behavior and will be published in due course. Furthermore, vaccine specificities like the number of peptides are likely to play a distinct role the quest optimality and therefore they have to be incorporated in the computer model as well.

## Figures and Tables

**Table 1:** Biological rules coding for interactions between cells or among cells and molecules and other specific mechanisms of the immune system. Each of the entries of this list corresponds to an algorithm implementing a specific activity of the immune cells.

Interactions	Activations
B phagocytosis of antigen	Activation of Mϕ
Mϕ phagocytosis of antigen	B cells anergy
DC phagocytosis of antigen	TH cells anergy
B presentation to TH	Priming of TH cells
Mϕ presentation to TH	TC cells anergy
DC presentation to TH	Activation of TC cells
Formation of immunocomplexes (IC)	
Mϕ phagocytosys	
Infection of EP cells	
Cytotoxicity of infected cells by TC	

Antigen ingestion and presentation	Other procedures

B exogenous pathway	Clone divisions
Mϕ exogenous pathway	Hematopoiesis
DC exogenous pathway	Plasma secretion of immunoglobulins
EP endogenous pathway	Entity movement
	Hypermutation of antibody

B: B cell, Mϕ: macrophage, DC: dendritic cell, TC: cytotoxic CD8+ T cell, Th: CD4+ T cell.
